# Structural Formation of Oil-in-Water (O/W) and Water-in-Oil-in-Water (W/O/W) Droplets in PDMS Device Using Protrusion Channel without Hydrophilic Surface Treatment

**DOI:** 10.3390/mi9090468

**Published:** 2018-09-14

**Authors:** Dong Hyun Yoon, Daiki Tanaka, Tetsushi Sekiguchi, Shuichi Shoji

**Affiliations:** 1 Faculty of Science and Engineering, Waseda University, 3-4-1, Okubo, Shinjuku-ku, Tokyo 169-8555, Japan; shojis@waseda.jp; 2 Research Organization for Nano & Life Innovation, Waseda University, 513, Tsurumaki-cho, Waseda, Shinjuku-ku, Tokyo 162-0041, Japan; d.tanaka@aoni.waseda.jp (D.T.); t-sekiguchi@waseda.jp (T.S.)

**Keywords:** microdroplet, double emulsion, three-dimensional structure, polydimethylsiloxane

## Abstract

This paper presents a simple method of droplet formation using liquids that easily wet polydimethylsiloxane (PDMS) surfaces without any surface treatment. Using only structural features and uniform flow focusing, Oil-in-Water (O/W) and Water-in-Oil-in-Water (W/O/W) droplets were formed in the full PDMS structure. Extrusion channel and three-dimensional flow focusing resulted in effective fluidic conditions for droplet formation and the droplet size could be precisely controlled by controlling the flow rate of each phase. The proposed structure can be utilized as an important element for droplet based research, as well as a droplet generator.

## 1. Introduction

Microdroplet technologies have been developed for quantitative volume control and the efficient isolation of samples in various chemical [1,2], biological [3,4,5,6], and material [7,8] research fields. The formation, mixing, and separation of droplets allow for the preparation, reaction, treatment, and analysis of specific samples.

In particular, droplet formation is the first step and is sometimes the final goal of all droplet-related research. Using immiscible liquids and specific methods, the liquid materials are isolated into individual droplets. Individual liquids from a dispersed phase drop off from the liquid lump into an immiscible surrounding fluid of a continuous phase that fills the gaps between droplets. Therefore, an external shear force from the surrounding fluid greater than the surface tension of the target liquid is essential for drop separation. Moreover, the target liquid should be detached from solid phase wall surfaces. These two conditions are fundamental requirements for droplet formation. 

In the field of microfluidics, various types of microchannels for droplet formation have been developed to obtain sufficient shear force from the carrier fluid because the importance of surface tension increases with scale-down [9,10]. T-shaped [11,12,13] or cross [14,15,16] channels are the conventional design for microdroplet formation and the size and generation frequency of the microdroplets are controlled by the flow rates of the injected liquids. 

However, contact of the liquid with channel walls is strongly dependent on the intrinsic wettability of the liquid; in this way, the type of droplet that can be formed is specified. Oil-in-water (O/W) droplets cannot be formed in a hydrophobic structure because the oil occupies the wall surface more readily than water. Therefore, suitable device materials or surface treatments have been considered according to the desired droplet types.

In contrast, double-layered droplets such as Water-in-Oil-in-Water (W/O/W) or O/W/O droplets also have an important role and provide more specific opportunities for many researchers [17,18,19]. For instance, the W/O/W double emulsion can isolate target materials in the oil shell, similar to a W/O single layer droplet. However, the minimized oil volume prevents the oil from influencing the inner target directly or the analysis results indirectly. Furthermore, some special organic solutions for the oil phase shell can allow for selective penetration of the droplet’s internal or external materials [20]. 

Double-layered droplets require a two-step droplet formation process. In the first step, a core droplet (inner phase) is formed, and then the core-embedded shell layer (middle phase) that was a carrier fluid in the first step is formed by the addition of carrier fluid (outer phase). Therefore, the two different liquids must contact the wall surface. However, as mentioned above, the two cases require opposite wetting conditions, thus many researchers have employed partial surface treatments and additional reagents to modify the wetting conditions [21,22,23,24,25,26,27]. 

Strong and uniform focusing flows in three-dimensional structures suppress undesired contact between the liquids and wall surfaces. Glass capillary based structures [28,29,30,31,32,33] have provided droplet formation with multiple layers, but the methods and materials are difficult to fabricate the device precisely, because the device requires skillful glass pulling, forging, and centering process. Moreover, the glass channels are low compatible with other fluidic elements and difficult to form complex channel network for highly functional systems.

In contrast, multi-layer polydimethylsiloxane (PDMS) devices also allow for the formation of double layered droplets and they are more easily integrated. However, Rotem et al. [34] discussed the limitations placed on the types of formable liquids, including contact angle or wettability on the solid structure. Thus, it seems that the structure requires the assistance of specific materials and techniques still. 

The most critical cause of insufficient formation results is the fact that wetting between the dispersed phase liquid and the wall surface stronger than the hydrodynamic shear or drag forces prevents the liquid from being isolated from the wall. In particular, non-flow areas at flat-end junctions lead to continuous spreading and flow of the dispersed phase liquid downstream along the surface without droplet formation. Furthermore, the partially modified two-dimensional focusing flow is not sufficient to resist these properties of the materials.

Hence, we propose a more efficient method for forming droplets of highly wetting liquids on a PDMS surface. Using functional structures and uniform three-dimensional focusing flow, droplets can be formed in the PDMS structure without any surface modification or treatment. This study is not only a technique for producing multi-phase and -layer droplets, but it also describes the development of a functional device element that is used in droplet based research.

## 2. Principle and Computational Analysis 

**Figure 1:**
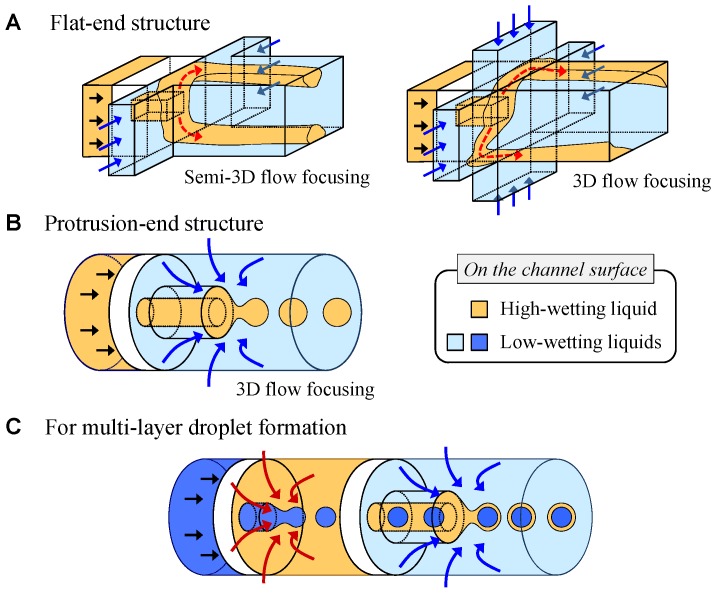
Principle of droplet formation for highly wettable liquid using protrusion channel and three-dimensional focusing.

To form droplets of a highly wetting liquid in a PDMS structure, the isolation of the liquid from the wall surface is the most critical and important step. For instance, oil phase liquids are highly wettable on the PDMS surface when compared to water phase liquids; the oil cannot be detached from the surface easily and form a droplet without a hydrophilic surface treatment. Therefore, stronger hydrodynamic forces or specific structures are necessary to prevent the oil from spreading or flowing along the surface. Conventionally, Romanowsky et al. [35] reported three-dimensional (3D) structures for working with double emulsions. They utilized a sandwiched focusing structure and a vertically high downstream channel for enhancing top- and bottom-side focusing and isolation. This combination achieved semi-3D focusing flow and it was possible to form droplets of some wettable liquids in the PDMS structure. However, depending on the materials and fluidic conditions, oil would still contact the top- or bottom-side wall surfaces. Therefore, oil droplets cannot be formed successfully, as shown in Figure 1A. In addition, even in the case of full 3D focusing flow, the oil may spread along the channel surface beside a low velocity area at the flat-end junction.

Therefore, we propose a protrusion-end structure to stably form oil phase droplets and prevent the oil from spreading by ensuring stronger wetting than flow focusing in the PDMS structure, as shown in Figure 1B. The protrusion structure is isolated from the wall and it avoids undesirable contact with the oil phase liquid on the surface and eliminates the zero velocity area at the junction of the immiscible liquids. Therefore, the functional structure allows for droplet formation from various liquids that are highly wettable on the device surface. Using this structure, multi-layered droplets can be also formed, as shown in Figure 1C. Furthermore, the three-dimensional channel structure guarantees more uniform and stable focusing to form droplets.

**Figure 2:**
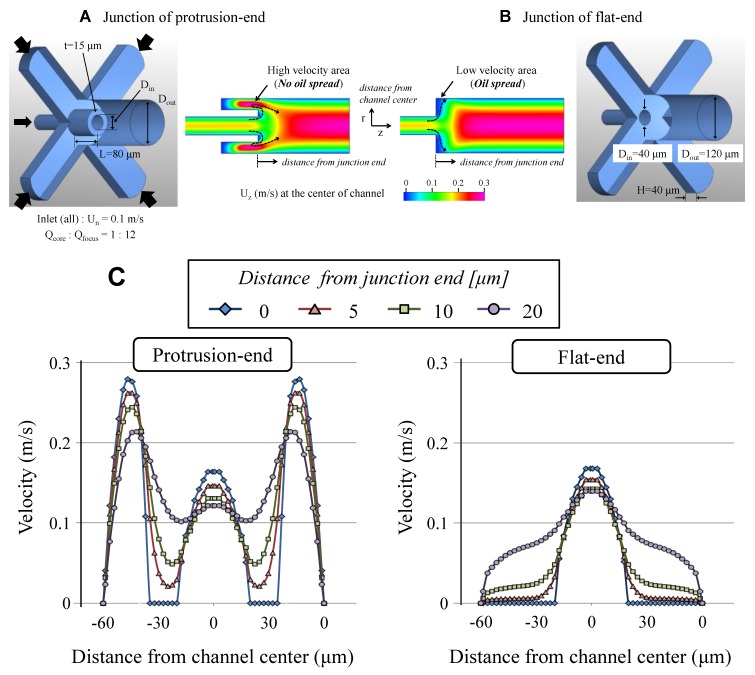
Flow distribution at the junction for droplet formation. (**A**) Protrusion-end structure; and, (**B**) Flat-end structure. (**C**) Velocity distributions in the axial direction at the junction area.

To verify the effects of the protrusion structure, we performed computational analysis (CFD-ACE+, ESI Group), as shown in Figure 2. The module used was “Flow” and the flow distribution in the structure under steady state was calculated. The working fluid was water for this analysis and it was injected from the normal direction into each inlet. Figure 2A,B shows structure designs and flow fields in the center of the structures and Figure 2C shows velocity distributions in the axial direction at the points away from junction-end. The flow rate of the focusing flow from the four branch channels is five times higher than the flow rate from the core channel.

As shown in the results, a low velocity area was formed near the junction of the core and focusing channels in the case of the flat-end junction. Thus, the high wetting liquid from the core channel spreads and flows onto the downstream wall along the area without hydrodynamic focusing or sweeping. 

In contrast, the protrusion-end increases the flow velocity next to the junction area. Therefore, the spreading and wetting of the liquid along the surface of the protrusion structure could be strongly suppressed by enhanced hydrodynamic forces. Consequently, it is possible to form droplets of the liquid, which wets more strongly on the channel surface than the continuous phase liquid, by the perfect isolation of the dispersed phase liquid from the wall.

## 3. Device Design and Fabrication

**Figure 3:**
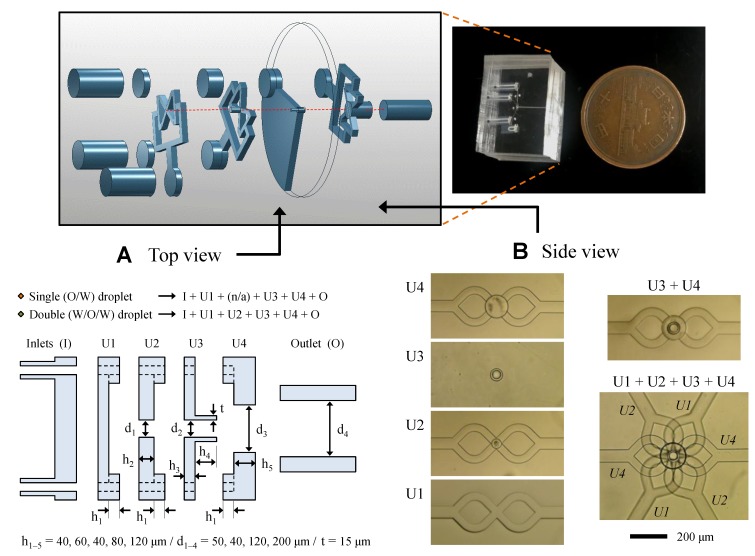
Images of three-dimensional device. (**A**) Schematic view and detailed dimensions of the units; (**B**) Microscopic images of the polydimethylsiloxane (PDMS) units and stacked structure.

The three-dimensional (3D) structure used to form O/W and W/O/W droplets was fabricated by stacking multiple PDMS units. Each unit was designed for uniform flow focusing and isolated tubular channels, as well as the introduction of each phase fluids. As shown in Figure 3, focusing channels, junctions, and the protrusion channel were formed in the units and they were combined to form a 3D structure. Three types of units were used to form oil phase (O/W) droplets and an additional unit was used to form W/O/W droplets.

In the case of the W/O/W droplet formation device, the first unit includes an injection channel and the second unit consists of two layers: a cylindrical junction layer and an injection layer consisting of four branch channels for uniform flow focusing. There are two layers in the third unit for a through-hole and a protruded tubular channel. Finally, the forth unit of two layers also consists of a four-branch focusing channel and a cylindrical through-hole. 

The units were formed by a PDMS molding process (SILPOT 184, Dow Corning Toray Co., Ltd., Tokyo, Japan) while using SU-8 molds (SU-8 3010/3025/3050, MicroChem Corp., Westborough, MA, USA) that were fabricated by a two-step photoresist patterning process. A PDMS guide film was employed to form penetrating structures and to successfully handle the thin unit during the molding process. After an oxygen plasma treatment on the surface of the PDMS units, the units were aligned and bonded under a microscope. Then, the guide film was removed and the next unit was bonded onto the previous units. Inlet and outlet layers were bonded onto the first and last units, respectively. Finally, additional PDMS was poured on the toppled structure to allow for clear observation and all PDMS devices were baked at 100 °C for at least one week to eliminate any effects of the plasma treatment and to obtain inherent hydrophobic surface.

Droplets of single (O/W) or double (W/O/W) layers were formed using water and mineral oil (M8410, CAS: 8042-47-5, SIGMA-Aldrich, St. Louis, MO, USA). To visualize the water phase flow, methylene blue that was dissolved in deionized water was introduced into the continuous phase for O/W droplets and the inner core phase for W/O/W droplets. Moreover, 1 wt% tween20 (P1379, CAS:9005-64-5, SIGMA-Aldrich, St. Louis, MO, USA) was employed as surfactant to prevent the merging of droplets downstream after formation.

All of the liquids were introduced into the device by syringes and syringe pumps (KDS210, kdScientific, Holliston, MA, USA). The droplet formation process was observed while using a high speed camera (FASTCAM-NEO, Photron, Tokyo, Japan). The size of the O/W or W/O/W droplets was evaluated by pixel counting and calculation from the captured images.

## 4. Results and Discussion

### 4.1. Oil-in-Water (O/W) Droplet Formation

**Figure 4:**
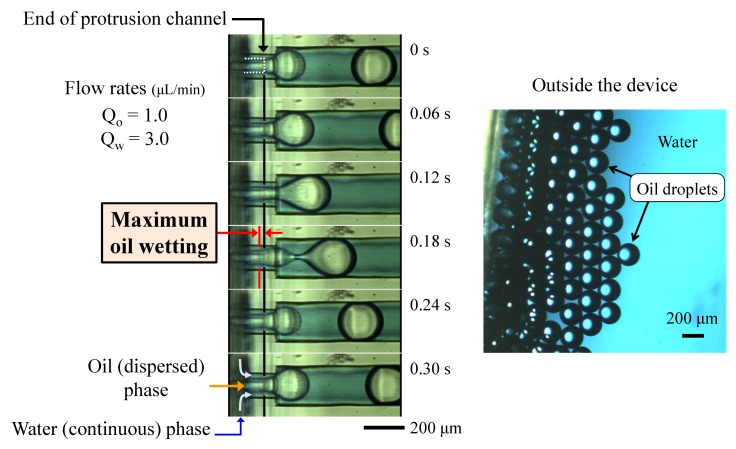
Visualization of the oil droplet (Oil-in-Water (O/W)) formation at the protrusion junction and the outside device.

The formation of oil droplets by water phase flow in a PDMS device is shown in Figure 4 (Supplementary Material, Video S1). At the end of the protrusion channel, the oil phase flow was focused by the water flow and was isolated from the wall surfaces. Therefore, the oil, which is highly wettable on PDMS surfaces, can be formed into dispersed droplets without continuous flow along the wall. The wetting of oil on the protrusion structure upstream was observed, but fully developed, continuous, high velocity flow beside the structure efficiently suppressed the wetting and spreading of the oil along the wall surface. Moreover, the droplets did not make contact with the downstream channel surface because of the continuous carrier flow, even when the droplet diameter was larger than the inner diameter of the downstream channel.

**Figure 5:**
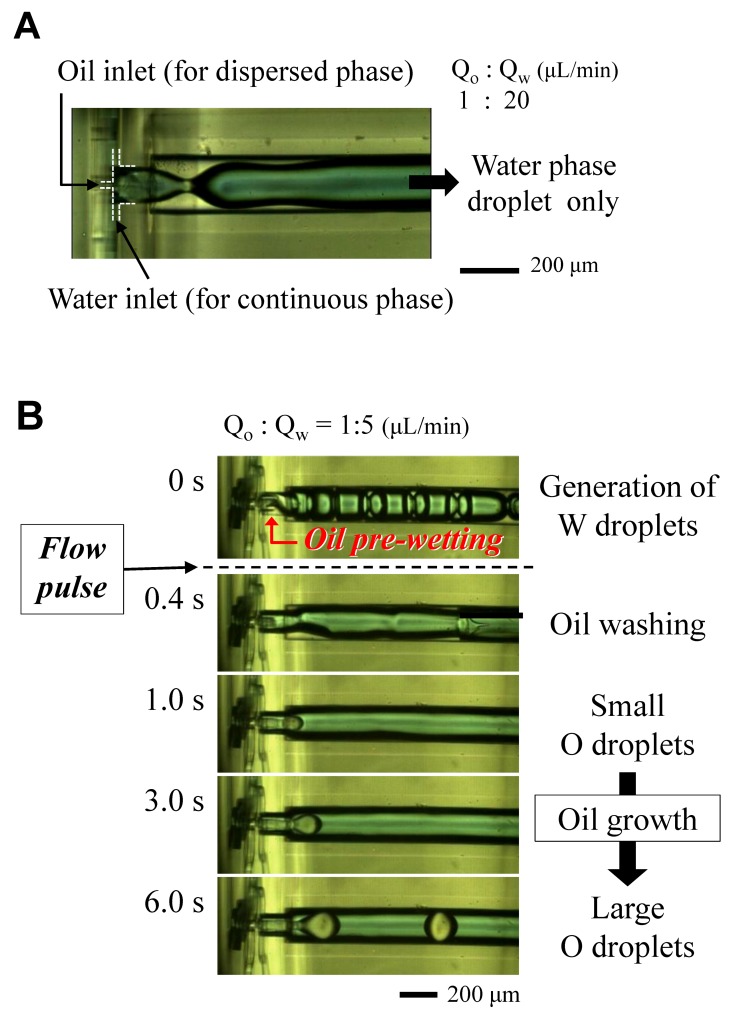
(**A**) Droplet formation result without protrusion structure; (**B**) Recovered formation of oil droplet via oil washing at the junction area with protrusion structure.

In contrast, Figure 5A shows the experimental result without the protrusion tip. In this case, we used an oil injection channel 20 μm in diameter, which is smaller than that of the protrusion tip, and a high ratio of oil phase and water phase flow rates. However, in this case, the water phase flow could not suppress the oil flow along the wall surface; thus, only water droplets were formed. 

When the oil was injected into the junction area before the water, our proposed device also allowed for the formation of water phase droplets only because of the dominant wall occupancy of the oil. However, the oil wetting can be washed by flowing water at a temporarily high flow rate, such as a short syringe push, as shown in Figure 5B (Supplementary Material, Video S2). The water flow did not concede the wall surface to the oil again after the washing. This result has been achieved in the protrusion-end device only, and this recovery is very useful for both end users and practical experiments.

**Figure 6:**
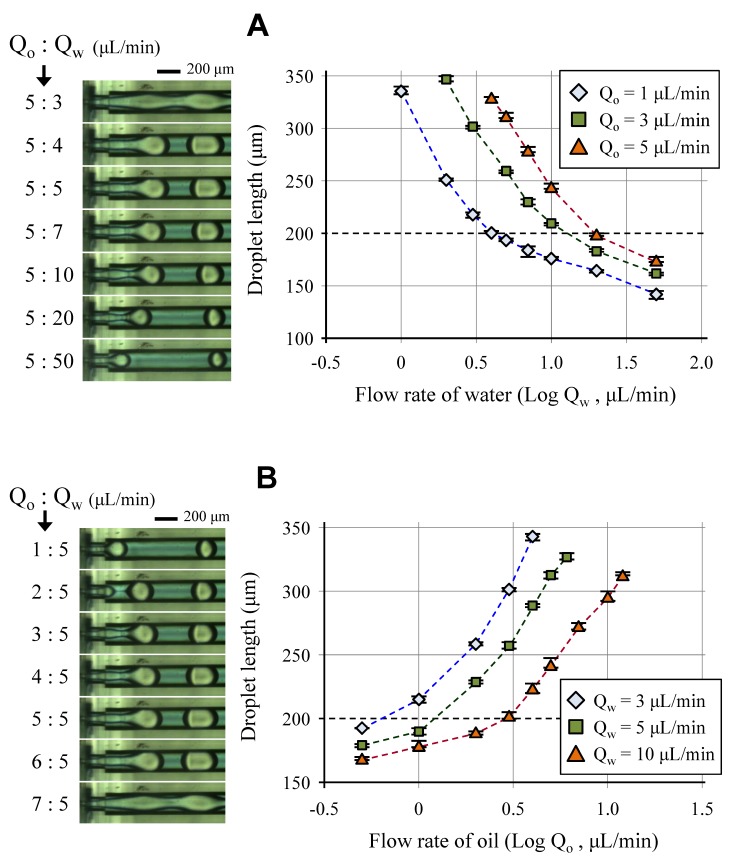
Control of the droplet size via flow rates of continuous phase flow (**A**) and dispersed phase flow (**B**).

The oil droplet size was controlled by adjusting the flow rates of each phase fluid, as shown in Figure 6. Similar to other droplet formation mechanisms, the droplet size was proportional to the flow rate of the dispersed phase fluid and inversely proportional to the flow rate of the continuous phase fluid. When the flow rate of the dispersed phase was about 1.5 times as large as the flow rate of the continuous phase, stable droplet formation in the dripping mode was changed to the unstable jetting mode or continuous flow. The droplet length ranged from 180 to 330 μm in the structure, and the standard deviation was smaller than 5% under the fluidic conditions of the stable dripping mode.

### 4.2. Water-in-Oil-in-Water (W/O/W) Droplet Formation 

**Figure 7:**
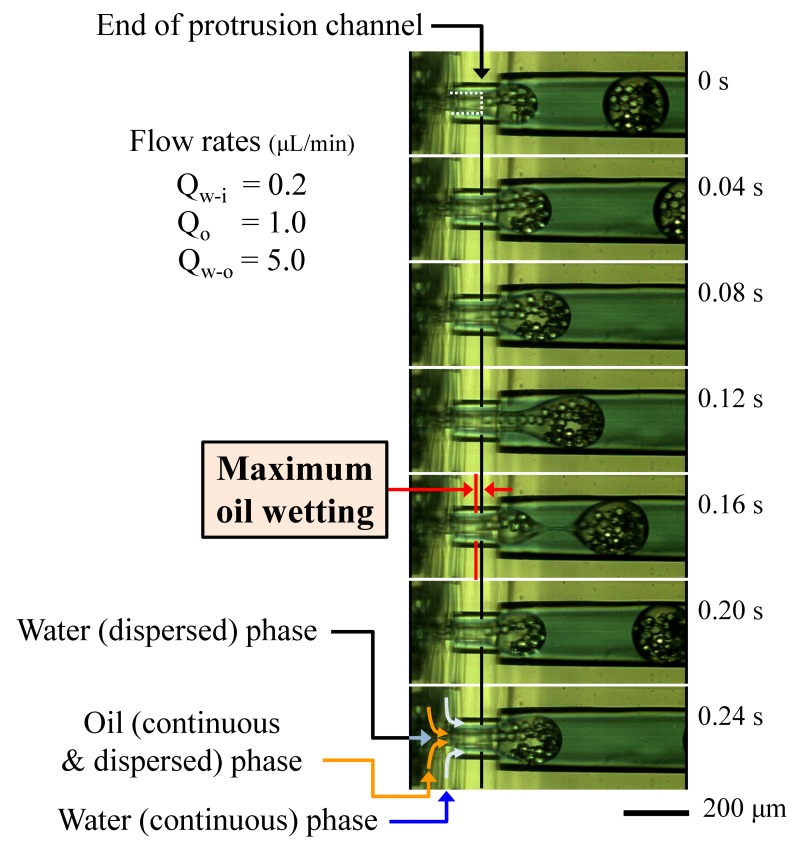
Visualization of the double layer (Water-in-Oil-in-Water (W/O/W)) droplet formation at the protrusion junction.

The proposed structure was applied to double emulsion (W/O/W droplet) formation, as shown in Figure 7 (Supplementary Material, Video S3). Water cores (W/O droplets) were formed at the first junction in the second unit (refer, Figure 3), then the oil droplets, including the water cores (W/O/W), were formed at the second junction (protrusion-tip). The oil droplets were successfully formed, even when the oil contained water phase droplets inside. In this case, the structure was efficiently prevented from contacting the oil and PDMS channel surfaces. Furthermore, the oil wetting upstream was suppressed by the continuous flow.

**Figure 8:**
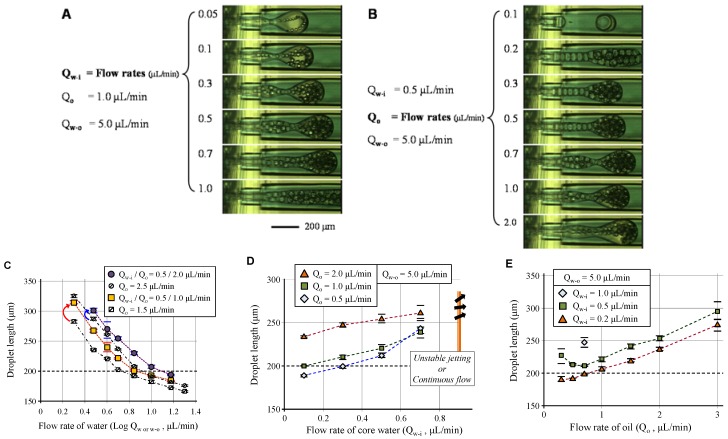
Control of the droplet size via flow rates of core phase flow (**A**,**D**), shell phase flow (**B**,**E**), and continuous phase flow (**C**).

The W/O/W droplet size was also controlled via controlling the flow rates of each fluid phase (Figure 8). Similar to the results of O/W droplet formation, the droplet size depended on the ratio between the oil phase flow containing a water core and the continuous outer flow of water phase. However, the inner water cores reinforced the oil layer from inside, thus the total size of the W/O/W droplets was larger than the O/W droplets, as shown in Figure 8C. Furthermore, the flow range that was capable of stable droplet formation was limited as compared to the case without inner cores, as shown in Figure 8D. Finally, Figure 8E shows the dramatic influence of the inner cores and oil on the droplet length. In the case where the flow rate of inner water was 0.5 μL/min, the droplet size first decreased and then increased with increasing oil flow rate. In the range of low oil flow rate, the size and density of the water cores were high. Therefore, the W/O/W droplet size decreased due to the reduced size and density of the water core, even though the volumetric oil flow rate increased. In contrast, when the core size and density deviated from the dominant range, the total droplet size increased with the flow rate of the oil phase. Finally, for a sufficiently low oil flow rate when compared to the inner water flow rate, small single-cored droplet formed (Figure 8B) due to the merging of water cores in the oil before the oil’s drop off. 

The O/W and W/O/W droplet formation results indicate that the proposed structure is similar to the co-flow model of droplet formation, because we used a sufficiently long protrusion-tip structure to prevent oil wetting and spreading. Therefore, the droplet size was highly dependent on the dimensions of the downstream channel, as well as the flow conditions.

It seems that the optimization of the protrusion-tip structure, including channel dimensions in each unit, is necessary to effectively utilize the shear force of the focusing flow. Moreover, the number and size of cores, such as a single core with thin shell, can be controlled by considering the channel dimensions. Furthermore, the number of water and oil layers can be increased by additional stacking of channel layers. 

For simple modeling, we proposed the channel structure of similar patterns and stacking that is shown in Figure 3. However, the most critical structure is the channel end that protrudes forward of the focusing channel to eliminate the low flow area where high wetting fluid spreads along the surface. Therefore, we believe that the functional pair structure (refer, U3 and U4 in Figure 3) can be integrated with any PDMS structures that can be manufactured.

## 5. Conclusions

We presented a method for forming single (O/W) or multi-layered (W/O/W) microdroplets while using an oil phase fluid in a PDMS structure. A protruded tubular end isolated from the wall surfaces combined with three-dimensional flow focusing allowed the formation of droplets from liquids that easily wet the PDMS surface without requiring any complex surface treatment. Furthermore, the efficient flow in the structure suppressed spreading of the liquid along the channel surface and washed off undesirable wetting of the liquid on the surface. Droplet size was controlled via the flow conditions and the number of droplet layers was increased via an increase in the channel layers. Multiple stacking of channel layers allowed for the formation of functional flows and the fabrication of precise structures. 

The proposed structure and method provide advanced droplet formation results compared to conventional methods using PDMS structures. Moreover, the method achieves results that were only possible on glass-based structures, while allowing for higher structural precision and fabrication reliability than the glass-based devices. Furthermore, the full PDMS structure can be stably integrated with other PDMS based functional elements that were already developed in the various fields without any critical limitations. Thus, we believe that the proposed method and structure have great potential for the enabling further droplet based chemical, material, or biological research.

## Supplementary Materials

The following are available online at http://www.mdpi.com/2072-666X/9/9/468/s1, Video S1: oil-in-water droplet formation at the protrusion structure. Video S2: recovered formation result of oil droplet via washing of oil wet on wall surface. Video S3: water-in-oil-in-water droplet formation at the protrusion structure.
